# Single nucleotide polymorphisms in a regulatory site of *VRN-A1* first intron are associated with differences in vernalization requirement in winter wheat

**DOI:** 10.1007/s00438-018-1455-0

**Published:** 2018-06-05

**Authors:** Nestor Kippes, Mohammed Guedira, Lijuan Lin, Maria A. Alvarez, Gina L. Brown-Guedira, Jorge Dubcovsky

**Affiliations:** 1 Department of Plant Sciences, University of California, Davis, CA 95616-8515 USA; 2 Present Address: Department of Plant Biology and Genome Center, University of California, Davis, CA 95616 USA; 3 Department of Crop Science, North Carolina State University, Raleigh, NC 27695 USA; 4 USDA-ARS Plant Science Research Unit, Raleigh, NC 27695 USA; 5 Howard Hughes Medical Institute, Chevy Chase, MD 20815 USA

**Keywords:** Wheat, Flowering, Vernalization, VRN1, RIP3, GRP2

## Abstract

Winter wheats require a long exposure to cold temperatures (vernalization) to accelerate flowering. However, varieties differ in the length of the period of cold required to saturate the vernalization response. Here we show that single nucleotide polymorphisms (SNP) at the binding site of the GRP2 protein in the *VRN-A1* first intron (henceforth, RIP3) are associated with significant differences in heading time after a partial vernalization treatment. The ancestral winter *VRN-A1* allele in ‘Triple Dirk C’ has one SNP in the RIP3 region (1_SNP) relative to the canonical RIP3 sequence, whereas the derived ‘Jagger’ allele has three SNPs (3_SNPs). Both varieties have a single *VRN-A1* copy encoding identical proteins. In an F_2_ population generated from a cross between these two varieties, plants with the 3_SNPs haplotype headed significantly earlier (*P* < 0.001) than those with the 1_SNP haplotype, both in the absence of vernalization (17 days difference) and after 3-weeks of vernalization (11 days difference). Plants with the 3_SNPs haplotype showed higher *VRN-A1* transcript levels than those with the 1_SNP haplotype. The 3_SNPs haplotype was also associated with early heading in a panel of 127 winter wheat varieties grown in three separate controlled-environment experiments under partial vernalization (36 to 54 days, *P* < 0.001) and one experiment under field conditions (21 d, *P* < 0.0001). The RIP3 polymorphisms can be used by wheat breeders to develop winter wheat varieties adapted to regions with different duration or intensity of the cold season.

**Electronic supplementary material:**

The online version of this article (10.1007/s00438-018-1455-0) contains supplementary material, which is available to authorized users.

## Introduction

Wheat is one of the most widely cultivated cereals and an important staple food worldwide. Almost 70% of 740M tonnes of annual production are used for direct human consumption making wheat yields crucial for human nutrition and global food security (FAOSTAT 2015). Recent estimates of the impact of climate change on crops yield have raised the interest in understanding how plant development is modulated by environmental cues (Cang et al. 2016; Cook et al. 2012; Franks et al. 2007; Liu et al. 2016).

Wheat varieties are divided into two major categories (winter and spring) based on their growth habit. Winter wheats require a long exposure to low temperatures (vernalization) to accelerate the transition from the vegetative to the reproductive phase. This requirement prevents exposure of delicate floral meristems to damaging freezing temperatures (Distelfeld et al. 2009a; Woods et al. 2016). By contrast, spring wheats have reduced or no vernalization requirement. Winter wheats are sown in fall in regions where wheat can tolerate winter freezing temperatures. However, where winter temperatures are too low, spring wheats are sown in the spring to avoid freezing. In Mediterranean regions, with mild and rainy winters, spring wheats are planted in the fall to take advantage of water availability during the winter.

Wheat varieties sown in these contrasting regions show different allele profiles at the main vernalization gene *VERNALIZATION1* (*VRN1*), a MADS-box transcription factor homologous to the meristem identity gene *APETALA 1* (*AP1*) in *Arabidopsis* (Yan et al. 2003; Trevaskis et al. 2003). Winter wheats carry the ancestral *VRN1* alleles, whereas spring wheats show deletions or mutations in regulatory regions located in the promoter or first intron. Changes in these regions have been observed in all three *VRN1* homologs (*VRN-A1, VRN-B1* and *VRN-D1*), as well as in the relatively recent duplicated paralog *VRN-D4* (Fu et al. 2005; Konopatskaia et al. 2016; Muterko et al. 2015, 2016; Yan et al. 2004a, 2003; Kippes et al. 2015; Chu et al. 2011; Pidal et al. 2009).

Additional differences in wheat vernalization requirement have been detected at the *VERNALIZATION2* (*VRN2*) and *VERNALIZATION3* (*VRN3*) loci. *VRN2* encodes a protein with a putative zinc finger and a CCT domain (ZCCT) that acts as a dominant long-day repressor of *VRN3* and flowering. *VRN2* deletions or loss-of-function mutations result in spring growth habit in diploid wheat and barley accessions (Yan et al. 2004b), but have not been observed so far in natural polyploid wheat species. Combination of deletions or non-functional *vrn2* alleles at all homologs in tetraploid and hexaploid wheat (e.g., by marker assisted selection) can generate spring wheats (Distelfeld et al. 2009b; Kippes et al. 2016).

During the fall, *VRN2* represses the expression of *VRN3* (= *FT1*), a homolog of the *Arabidopsis* flowering promoter *FLOWERING LOCUS T* (*FT*) (Yan et al. 2006). *FT* encodes a mobile protein that travels from the leaves to the apex (Corbesier et al. 2007; Tamaki et al. 2007) where it forms a complex with FD-like and 14-3-3 proteins. This complex binds to the *VRN1* promoter and induces flowering (Taoka et al. 2011; Li et al. 2015). During the winter, a slight induction of *VRN1* is sufficient to repress *VRN2*, which favors *FT1* up-regulation when days get longer during spring (Chen and Dubcovsky 2012). These interactions result in a positive feedback-loop that irreversibly promotes wheat flowering in the spring (Loukoianov et al. 2005).

The natural allelic variation in *VRN-A1* responsible for differences between spring and winter wheats is well characterized. However, less is known about the role of *VRN-A1* on the differences among winter wheats in the duration of the cold period required for saturating the vernalization response. Three independent studies found that a large proportion of the variation in vernalization requirement among winter wheat varieties is linked to the *VRN-A1* locus. However, the three studies propose alternative explanations for these differences. Diaz et al. (2012) suggested that the differences were caused by the presence of a single copy of *VRN-A1* in ‘Claire’ and three in ‘Hereward’. Li et al. (2013) argued that the differences in heading time were caused by an amino acid polymorphism at position 180 between ‘Jagger’ (alanine) and ‘2174’ (valine). More recently, Kippes et al. (2015) found that both ‘Claire’ and ‘Jagger’ also differed from ‘Hereward’ and ‘2174’ by single nucleotide polymorphisms (SNPs) at the binding site of a GLYCINE RICH RNA-BINDING PROTEIN 2 (GRP2) in a region of the *VRN-A1* first intron designated as RNA Immune Precipitation fragment 3 (RIP3, Xiao et al. 2014 and; Kippes et al. 2015). RIP3 natural polymorphisms were shown to disrupt the binding of GRP2 to the RIP3 site (Kippes et al. 2015), but their effects on *VRN1* expression and heading time were not characterized. No variation in the RIP3 site was detected in the *VRN-B1* or *VRN-D1* genes (Kippes et al. 2015).

In this study, we show that polymorphisms in the *VRN-A1* RIP3 region segregating in an F_2_ population are associated with differences in *VRN-A1* transcript levels and heading time independently of differences in *VRN-A1* copy number and protein sequence. We also show that polymorphisms in the RIP3 region in a panel of winter wheat varieties from different geographic regions are associated with differences in heading time. The distribution of RIP3 alleles and *VRN-A1* copy number among winter wheat varieties and the utilization of these variants in winter wheat breeding are discussed.

## Materials and methods

### Plant material

The parental lines of the F_2_ population analyzed in this study were the winter wheat lines Triple Dirk C (TDC) and CS5402 (Kippes et al. 2014, 2015). TDC is part of a set of near isogenic lines for vernalization alleles described in Pugsley 1971 and 1972. CS5402 is a substitution line of chromosome 5D of Chinese Spring (dominant *Vrn-D1* allele) by the *Ae. tauschii* chromosome 5D (recessive *vrn-D1* allele). Both lines have a single copy of the *VRN-A1* gene homozygous for the recessive *vrn-A1* allele, which encodes for identical VRN-A1 proteins (alanine residue at position 180, Li et al. 2013). The two accessions differ in the sequence of the RIP3 region in intron 1. Relative to the RIP3 canonical sequence, the *VRN-A1* haplotype from TDC has one SNP (henceforth 1_SNP, GenBank AY747600) and the one from CS5402 three SNPs (henceforth 3_SNPs, GenBank KR422423, Kippes et al. 2015).

For the association study, we used a panel of 127 winter lines from the USDA National Germplasm Collection from diverse geographical origin. Sixty-five percent of the lines were collected from 31 different countries in Europe, 15% from 9 countries in Asia, 18% from 4 countries in the Americas, and 1% from Australia. These 127 accessions were selected from a larger set after filtering out accessions carrying alleles associated with a spring growth habit using available molecular markers (Yan et al. 2004b, 2006; Fu et al. 2005).

### Molecular markers and *VRN1* polymorphisms

Genomic DNA was isolated from individual plants for the F_2_ study and from bulks of leaves from four seedlings of each accession for the winter wheat panel (MAG-Bind Plant DNA Plus 96 kit, Omega Bio-Tek, Norcross, GA). Kompetitive Allele Specific PCR (KASP) fluorescent assays were used to detect polymorphism among the 127 accessions at wheat vernalization loci. PCR was run according to manufacturer’s instructions, using a reaction volume of 4 µL, which consisted of 2 µL 2× KASPar reaction mix, 0.05 µL 72× assay mix, and 2 µL of template DNA (10 ng µL^−1^). Endpoint genotyping was conducted using the software KlusterCaller (LGC Genomics, Hoddeson, UK).

Assays developed from published sequences of causal genes were used to distinguish lines in the core collection possessing spring alleles at *Vrn-A1, Vrn-B1, Vrn-D1*, and *Vrn-B3* loci (Supplemental Table 1). The exception was the KASP assay wMAS000033 used for detection of the *Vrn-A1a* spring allele developed from the contextual sequences of iSelect SNP marker IWA0001 determined to be associated with *Vrn-A1a*. Accessions carrying alleles for spring growth habit at any *VRN* locus were excluded from further analysis. The *VRN-A1* RIP3 region was sequenced in the 127 winter accessions by Sanger sequencing using primers listed in Table 1. Genotyping of the RIP3 alleles in the F_2_ population was conducted with a KASP assay (Table 1) run on a 7500 Fast Real-Time PCR system (Applied Biosystems). *VRN-A1* copy number was determined using a TaqMan assay from four biological replications per genotype as described in Kippes et al. (2015). Differences in flowering time between *VRN-A1* copy number classes were analyzed using Tukey’s test (*P* < 0.05). The *VRN-A1* promoter from Triple Dirk C (TDC) was sequenced by Sanger sequencing (GenBank MH347747) using four overlapping PCR products amplified with primers listed in Table S3.

**Table 1:** List of primers used for RIP3 characterization and *VRN-A1* transcripts abundance by RT-qPCR

Use	Name	Primer sequence (5′–3′)
RIP3 A genome sequencing	RIP-3_F1	AATCACACCTCAGGATTTCAT
	RIP-3_R1	GATGGGTCATAAGGTTTTGC
RIP3 A genome Kasp assay^a^	1_SNP_F	TCTCACAGTCATTGTTGTTGGTATG
	3_SNPs_F	TCTCACAGTCATTGTTGTTGGTATC
	Reverse	AGCAATCAAGTTGTAACATAAATAATTA
*VRN1 RT-qPCR full transcript*	q.VRNA1-L-F1	TCCACCGAGTCATGTATGGA
	q.VRNA1-L-R1	GAGAACCTTTTCTGCATAAGAA
*VRN1 RT-qPCR short transcript*	q.VRNA1-S-F1	CACCAAGGGAAAGCTCTACG
	q.VRNA1-S-R1	GTTAACTTGTAACTGGGAGCTAA

^a^KASP assay primers do not include tail sequences

### Principal component analysis

Accessions from the winter panel were previously genotyped with the Illumina Infinium 9K iSelect platform as part of the characterization of the NSGC core collection of winter and facultative common wheat (Bonman et al. 2015). Genotypes of 4,483 markers for all accessions were obtained from The Triticeae Toolbox (https://triticeaetoolbox.org/wheat). Principal component analysis was conducted in Tassel v5.0 (http://www.maizegenetics.net/tassel) using the covariance matrix for markers having minor allele frequency greater than or equal to 0.05. Lines missing more than 10% of data were removed.

### RT-qPCR

The last expanded leaf of four biological replications per genotype was collected in liquid nitrogen, and RNA was extracted using the Spectrum Plant Total RNA Kit (Sigma-Aldrich). RNA samples were treated with DNase I (RQ1 RNase-Free DNase, Promega) and first strand cDNAs were synthesized from 1 µg of total RNA using the High Capacity Reverse Transcription Kit (Applied Biosystems). Quantitative RT-PCR was performed using SYBR Green and a 7500 Fast Real-Time PCR system (Applied Biosystems). *ACTIN* was used as an endogenous control. Primers are detailed in Table 1; primers for *ACTIN* were previously described (Dubcovsky et al. 2006). Transcript levels were expressed as linearized fold-*ACTIN* levels calculated by the formula 2^(*ACTIN* CT − *TARGET* CT)^ ± standard error (SE) of the mean. The resulting number indicates the ratio between the initial number of molecules of the target gene and the number of molecules of *ACTIN*.

### Phenotyping

#### Vernalization

Seeds were germinated in 2 × 2 plastic inch pots filled with vermiculite in a greenhouse with 16-h light and 8-h dark photoperiod. One week after planting, seedlings were moved to a growth chamber for 3 weeks of vernalization at 4 °C under 16-h light/8-h dark photoperiod. Similar conditions were used in the facilities at the University of California, Davis (UCD) and North Carolina State University (NCSU).

After vernalization, up to 10 seedlings were transplanted into 0.7 L black plastic containers (Stuewe and Sons, Tangent, OR) with 1:1 Metro Mix 2 and soil. Two grams of slow release fertilizer (Multicote 14–14–16, N-P-K) was incorporated with the soil in each cone. Cones were placed in greenhouses or growth chambers under the conditions described below.

#### Growth chamber

After vernalization, four replications of each entry were grown in a controlled-environment chamber at NCSU in a completely randomized design. Plants were grown under 20/18 °C day/night temperature and 16-h light/8-h dark photoperiod. Heading date was noted as the number of days after transplanting when the spike fully emerged from the boot (Zadoks 60, Zadoks et al. 1974). The experiment was ended 175 days after planting.

#### Greenhouse

After the vernalization treatment described above, plants were grown at greenhouse facilities at UCD (four replications) and NCSU (up to six replications) in a completely randomized design. Supplemental lighting was used to provide 16-h light/8-h dark photoperiod. During the growth period, the minimum temperatures during the night varied from 10 to 18 °C and the maximum temperatures during the day ranged from 21 to 32 °C. The experiment was ended 210 days after planting. Heading date is reported as the number of days after transplanting when the spike fully emerged from the boot (Zadoks 60, Zadoks et al. 1974).

#### Field

Three grams of seed of each entry were sown in a single 1.5 m row at the NCSU Research Farm at Raleigh, NC (35.73°N, 78.68°W; elevation: 116.5 m) on October 17, 2015. Heading date was recorded as the number days after January 1st, when 50% of the plants in a plot were at Zadoks 60 growth stage.

## Results

### The RIP3 region at the first intron of *VRN-A1* is associated with differences in flowering time

To determine the effect of the two *VRN-A1* RIP3 natural alleles on heading time, we intercrossed TDC (1_SNP allele) with CS5402 (3_SNPs allele). These two lines have a single *VRN-A1* copy encoding identical proteins. We generated an F_2_ population of 142 plants segregating for these haplotypes, genotyped them for the RIP3 alleles and recorded heading time under three different vernalization treatments (no vernalization, 3 weeks vernalization and 7 weeks vernalization).

**Fig. 1:**
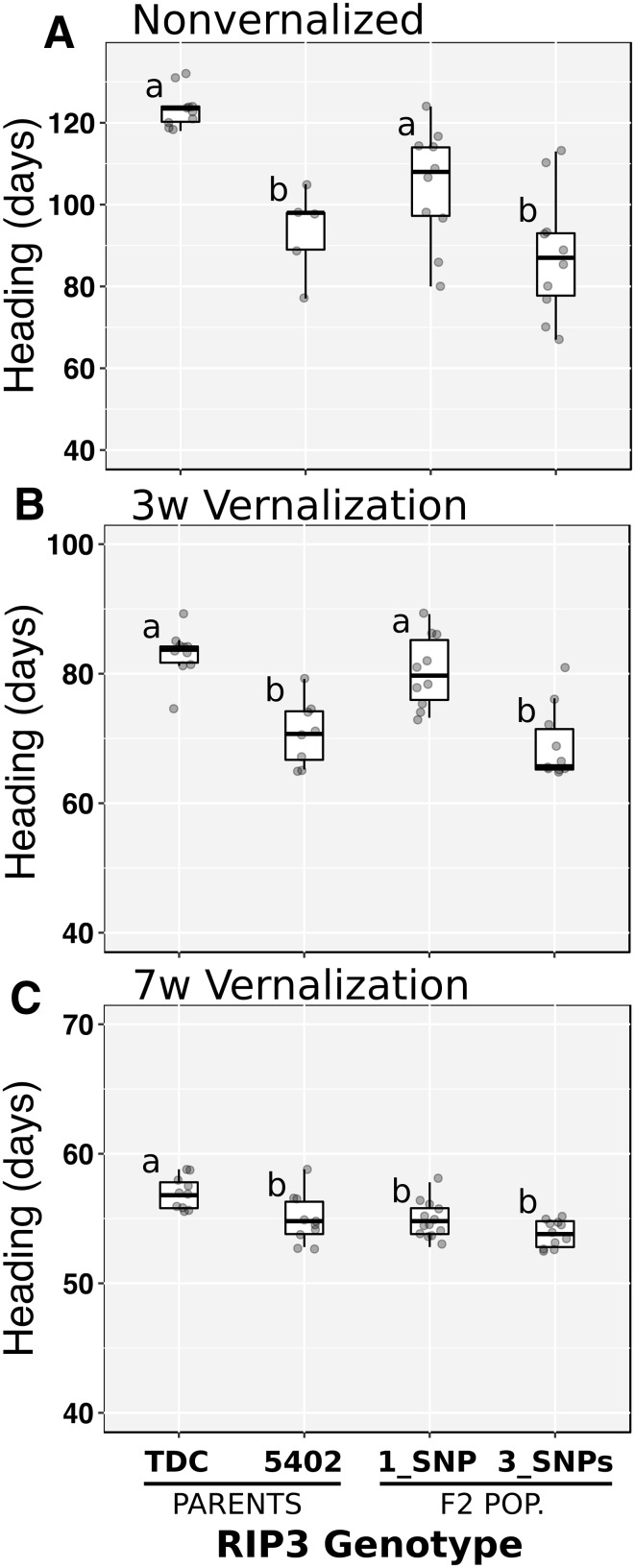
Difference in heading time between TDC (1_SNP) and CS5402 (3_SNPs) and between F_2_ plants homozygous for the RIP3 haplotypes. Data represents heading time means of at least eight plants per genotype under three different treatments (no vernalization and 3 or 7 weeks of vernalization). Different letters indicate significant differences (Tukey’s test *P* < 0.05)

In the absence of vernalization, the parental line CS5402 headed 30.2 days earlier than TDC, whereas F_2_ plants homozygous for the 3_SNPs haplotype flowered 16.9 days earlier than plants homozygous for the 1_SNP haplotype (*P* < 0.0001, Fig. 1a). When plants were vernalized for 3 weeks, the difference in heading time between parental lines was reduced to 12 days (TDC later than CS5402) and those between F_2_ homozygous plants to 11.3 days (1_SNP haplotype later than 3_SNPs haplotype, *P* < 0.0001, Fig. 1b). After 7 weeks of vernalization, the difference in heading time between the parental lines was reduced to only 2 days (TDC later than CS5402, *P* = 0.0128) and those between homozygous F_2_ plants for the two RIP3 homozygous plants were no longer significant (0.9 d, *P* = 0.0578). These results show that there are significant differences in heading time linked to polymorphisms at the RIP3 intronic region.

### Flowering time differences are linked to differences in *VRN-A1* expression levels

To study the effects of RIP3 polymorphisms on *VRN-A1* transcript levels, we sampled plants from the F_2_ population homozygous for the 1_SNP and 3_SNPs haplotypes at different time points of the partial vernalization treatment. Leaf samples were collected from 3-week-old plants immediately before vernalization (3w), 48 h after the plants were transfer to 4 °C (48 h), after 1 and 3 weeks of vernalization (1wV and 3wV) and 3 weeks after the plants were returned to room temperature (3wR).

**Fig. 2:**
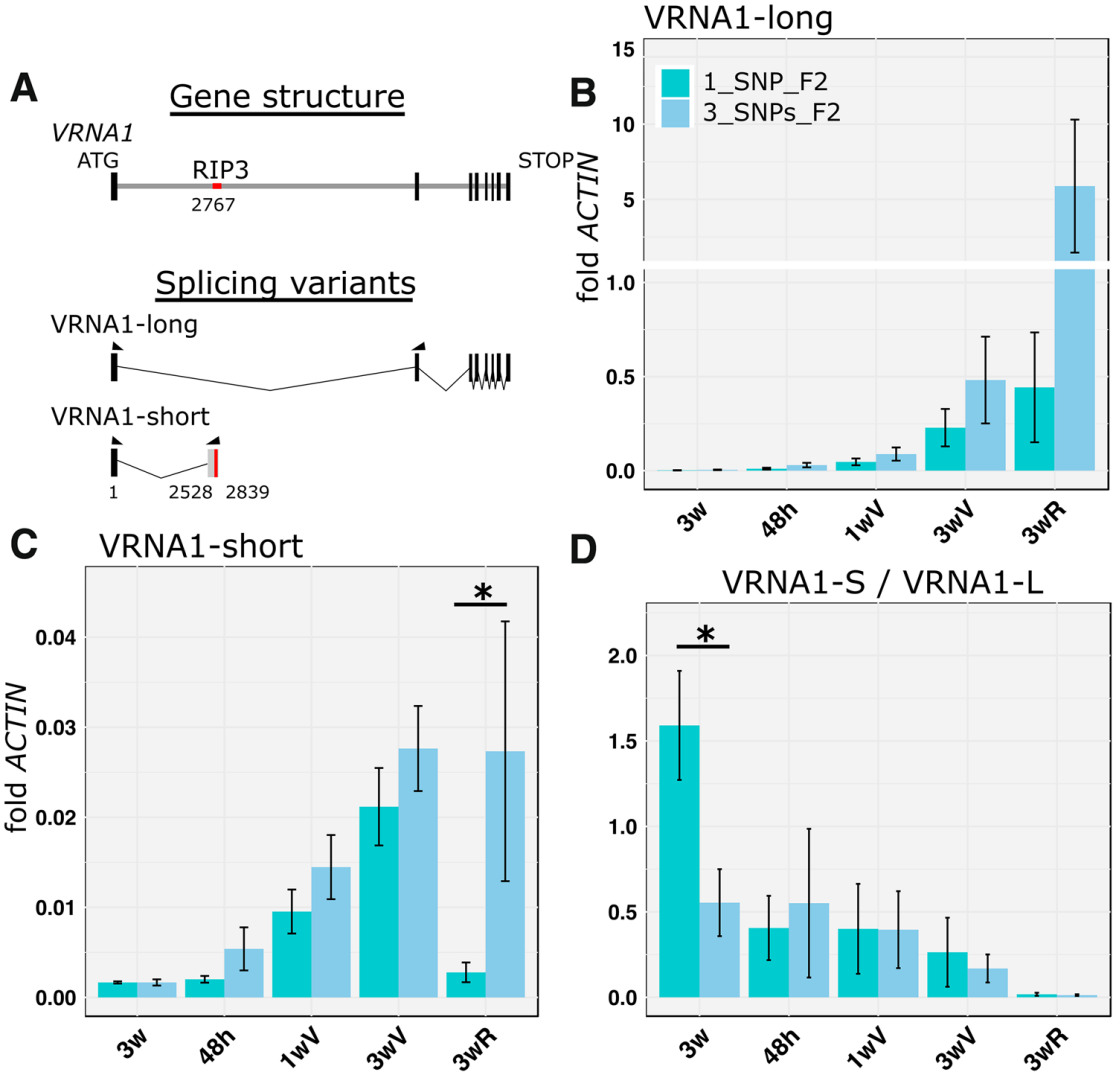
Transcript levels of *VRN-A1* alternative splice variants during vernalization. Transcript levels of VRN-A1 alternative splice variants were studied in F_2_ plants homozygous for the two RIP3 haplotypes. Leaf samples were collected from 3-week-old plants immediately before vernalization (3w), 48 h after the plants were transfer to 4 °C (48 h), after 1 and 3 weeks of vernalization (1 and 3wV) and 3 weeks after the plants were returned to room temperature (3wR). (A) Schematic representation of the different *VRNA1* transcripts studied. Arrowheads indicate regions complementary to the qRT-PCR primers utilized. The *VRN-A1* alternative splice variant includes the RIP3 region (red). *VRNA1*-long correspond to the complete gene (B) and *VRNA1*-short to the alternative splice variant (C). Average ratios of short/long transcript versions are presented in D. Bars represent means of four biological replications and error bars correspond to SEM (* *P* < 0.05)

Since two alternative splice variants of *VRN-A1* were detected in the study of Xiao et al. (2014), we designed specific primers to amplify each variant separately. The long *VRN-A1* transcript variant (henceforth, long variant) encodes the complete gene, whereas the short *VRN-A1* transcript variant (600 bp, henceforth, short variant) includes the complete first exon (185 bp) and a small portion of the first intron. The short transcript ends a few base pairs downstream of the RIP3 region, located 2,767 bp downstream of the *VRN-A1* start codon in TDC (Fig. 2a). Figure 2b presents the transcript levels of the long variant, Fig. 2c the short variant, and Fig. 2d the ratio between the two splice variants.

For the *VRN-A1* long variant, the transcript levels of the F_2_ plants carrying the 3_SNPs haplotype were 2- to 13-fold larger than those of plants carrying the 1_SNP haplotype. However, the differences were not significant at any of the time points or in the combined repeated measurements ANOVA (*P* = 0.18, Fig. 2b).

For the *VRN-A1* short variant, transcript levels of the F_2_ plants carrying the 3_SNPs haplotype were significantly higher than those of plants carrying the 1_SNP haplotype in the combined repeated measures ANOVA (*P* = 0.0003). However, for the individual time points the difference between haplotypes was significant only at 3 weeks after vernalization (*P* = 0.0177, Fig. 2c 3wV).

The ratio between the short and long *VRN-A1* variants showed a decrease during and after vernalization (Fig. 2d). This was the result of faster increases of the long variant relative to the short variant (Fig. 2d). After vernalization, when the plants were returned to room temperature, the short/long variant ratio was from 40- to 60-fold smaller than the same ratio before vernalization (Fig. 2d), suggesting a decreasing importance of the short variant at this time point. Before vernalization, the ratio between the short and long *VRN-A1* variants was three times higher in the plants carrying the 1_SNP RIP3 haplotype than in those carrying the 3_SNPs haplotype (Fig. 2d, P = 0.0462). No significant differences between haplotypes were detected for the other time points (Fig. 2d).

### Frequency and effect of the RIP3 haplotypes on heading time in a winter wheat panel

**Fig. 3:**
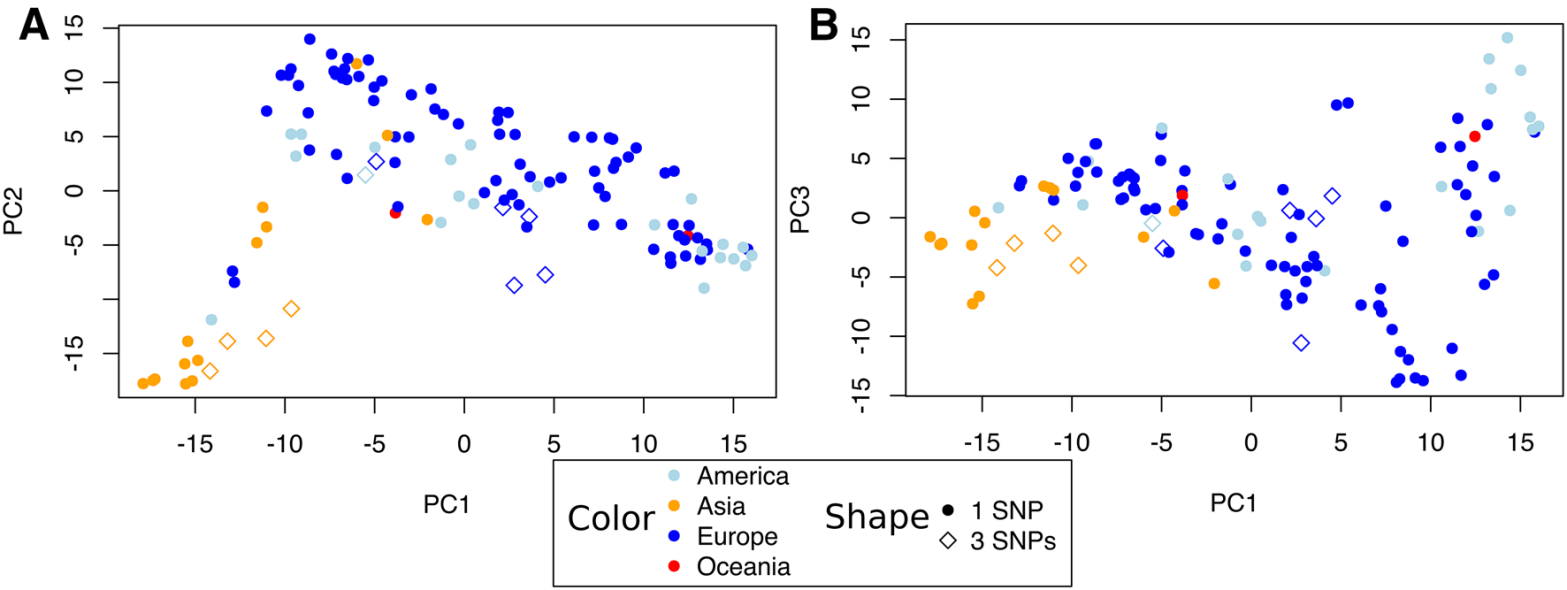
Principal component analysis (PCA) of the winter panel used in this study. The PCA was based on 4483 SNP markers. Colors indicate geographical origin and RIP3 genotypes are indicated by circles (1_SNP) or open diamonds (3_SNPs). **a** First (PC1) and second (PC2) principal components. **b** First (PC1) and third (PC3) principal components

Based on the observed effect of the RIP3 haplotypes on heading time in the F_2_ population, we decided to study their effect in a panel of 127 winter lines from diverse geographical origins. The 1_SNP haplotype was found in 90.5% of the accessions whereas the 3_SNPs haplotype was found in only 9.5% of the accessions. A principal component analysis showed that lines with the 3_SNPs haplotype tend to cluster together in two separate groups of Asian or European origin (Fig. 3, Supplemental Table S2).

**Fig. 4:**
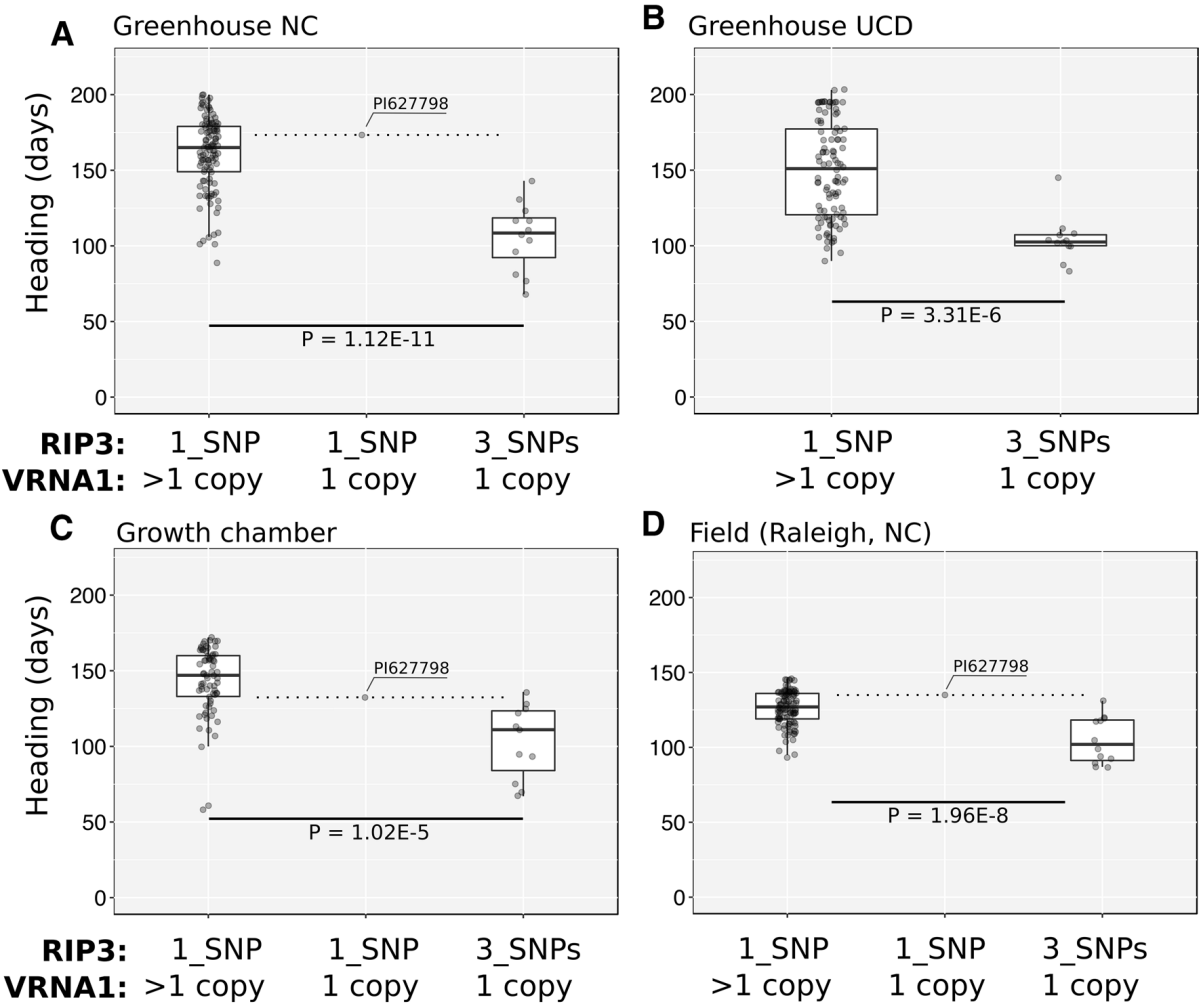
Heading time differences between RIP3 haplotypes in a winter wheat panel. Independent experiments were conducted under **a**–**c** control environmental conditions (3-week vernalization, or **d**) under natural vernalization in the field (Raleigh, NC). **a, b** Plants transferred to greenhouses at UCD and NCSU after 3-weeks vernalization. **c** Plants were vernalized in a growth chamber at 4 °C for 3 weeks and then temperature settings were switched to warm conditions (20/18 °C day/night) until heading

To study the effect of the RIP3 haplotypes on heading time the winter wheat panel was grown in three independent experiments with partial vernalization conditions and one field experiment. The first experiment was conducted in a grown chamber, where plants were first exposed to 3 weeks of vernalization and then moved to room temperature conditions. In two additional experiments performed at the University of California Davis (UCD) and North Carolina State University (NCSU), plants were transferred to greenhouses after 3 weeks of vernalization. The final experiment was conducted under field conditions in Raleigh, North Carolina. In the three experiments grown under controlled-environments, plants carrying the 3_SNPs haplotype flowered significantly earlier (36–54 days, *P* < 0.0001) than plants with the 1_SNP haplotype (Fig. 4a–c; Table 2). In the field experiment, plants with the 3_SNPs haplotype flowered 21 days earlier than the plants with the 1_SNP haplotype (Fig. 4d; Table 2).

**Table 2:** ANOVA for heading time of winter wheat lines carrying different RIP3 haplotypes

	GH^a^ NC	GH^a^ UCD	Growth chamber^b^	Field^c^
3 SNPs	106.1 ± 2.3	104.3 ± 3.2	105.8 ± 2.7	104.8 ± 1.1
1 SNP	161.0 ± 6.5	150.1 ± 4.4	142.6 ± 7.6	126.4 ± 4.5
Dif. (days)	54.9	45.8	36.8	21.6
*P* value	1.11E-11	3.31E-06	1.01E-05	1.95E-08

^a^GH = Greenhouse after 3-weeks vernalization

^b^Growth chamber after 3-week vernalization

^c^Field conditions after natural vernalization (Raleigh, NC)

### Variation in *VRN-A1* copy number

To study the effect of *VRN-A1* copy number variation on heading time we characterized all lines in the winter wheat panel with a TaqMan assay developed by Diaz et al. (2012). *VRN-A1* copy number varied from one (10.2% of the lines) to four copies (3.9% of the lines), with most lines carrying two (35.4%) or three copies (50.4%). Most of the winter lines with a single *VRN-A1* copy have the RIP3 3_SNPs haplotype. The only exception was PI 627798, which has a single *VRN-A1* copy and the 1_SNP haplotype (same as TDC). PI 627798 heading time was more similar to plants with the 1_SNP haplotype and multiple *VRN-A1* copies, than to plants with the 3_SNPs haplotype and a single *VRN-A1* copy. Compared with the plants in the last class, PI 627798 was the latest flowering in two experiments (Fig. 4a, d) and the second latest in the remaining one (Fig. 4c, not tested in b). These results are consistent with the late flowering of the plants carrying the 1_SNP haplotype in the F_2_ population.

We detected no significant differences in heading time among varieties carrying two, three or four copies of *VRN-A1* in any of the three pairwise comparisons. Taken together, these results suggest that *VRN-A1* copy number variation has limited effect on heading time under the conditions used in our four experiments.

## Discussion

### *VRN-A1* is linked to differences in vernalization requirement in winter wheat

The length of the vernalization period required to saturate the acceleration of flowering varies widely among winter wheat varieties. Some varieties reach the saturation point after only 3 weeks of vernalization (sometimes called ‘facultative’ types), but most varieties require approximately 6 weeks of vernalization to reach this point. In some exceptional cases, vernalization treatments of up to 8 weeks are necessary to saturate the acceleration of flowering (Brooking 1996; Kosner and Pankova 2002).

The genetic factors controlling differences in vernalization requirement among winter varieties are not as well understood as those controlling differences between winter and spring varieties (Distelfeld et al. 2009a). However, studies that genetically mapped genes controlling heading time in winter wheat using partial vernalization treatments (3–4 weeks of cold treatment) found that at least part of these differences were linked to the *VRN-A1* locus (Diaz et al. 2012; Li et al. 2013). A similar conclusion is supported by this study.

One limitation of linkage studies using small segregating populations is that they cannot rule out the possibility of other linked SNP or genes affecting the trait. This is particularly critical for the *VRN-A1* locus that is tightly linked with the *PHYC* gene (0.02 cM, Yan et al. 2003). *PHYC* mutants affect heading time in wheat (Chen et al. 2014) and natural variation in this gene is associated with variation in flowering time in *Arabidopsis* (Balasubramanian et al. 2006) and pearl millet (Saïdou et al. 2014). Fortunately, Li et al. (2013) were able to find recombination events between *VRN-A1* and *PHYC* that demonstrated that the differences in vernalization requirement between their parental lines ‘Jagger’ and ‘2174’ were linked to *VRN-A1* and not to *PHYC*.

### Different polymorphisms in *VRN-A1* may contribute to flowering time differences

Although there is agreement on the contribution of *VRN-A1* to the differences in vernalization requirement among winter wheats, there is no agreement on the current interpretation of the causal polymorphisms. Diaz et al. (2012) suggested that the differences in heading time linked to *VRN-A1* were caused by differences in *VRN-A1* copy number among Claire (1 copy), Malacca (2 copies) and Hereward (3 copies). However, the F_2_ population they generated from the cross between Claire and Hereward segregated also for the Ala/Val polymorphism at position 180 of the VRN-A1 protein and for the 3_SNPs/1_SNP polymorphism in the RIP3 site of the first intron, complicating the interpretation of these results.

Diaz et al. (2012) also analyzed a double haploid population from the cross between Malacca and Hereward, and found that the plants carrying three *VRN-A1* copies tended to head later than the plants carrying two *VRN-A1* copies after 4 weeks of vernalization. In this population, both parental lines have the valine residue at position 180 and the 1_SNP haplotype at the RIP3 site, increasing the chances that the observed differences in heading time were caused by the differences in *VRN-A1* copy number. Similarly, Guedira et al. (2016) observed in a RIL population from a cross between cultivars 26R61 and AGS 2000, both having the Val 180, that after 2 or 4 weeks of vernalization lines having three *VRN-A1* copies flowered later than those having two *VRN-A1* copies. However, it is not possible to rule out completely the effect of a linked gene given the small size of these segregating populations.

Li et al. (2013) proposed that the early heading time observed after partial vernalization of the plants carrying the Jagger *VRN-A1* allele (Ala 180) relative to 2174 (Val 180) were caused by different amino acid residues at position 180. However, these two winter wheat varieties differed also in *VRN-A1* copy number (Jagger one *VRNA1* copy *vs*. 2174 two *VRNA1* copies) and the RIP3 haplotype in the first intron (Jagger 3_SNPs *vs*. 2174 1_SNP), complicating the interpretation of the results.

The *VRN-A1* allele in CS5402 is almost identical to the alleles present in Jagger and Claire, all carrying a single *VRN-A1* copy, the 3_SNPs RIP3 haplotype and Ala 180. Therefore, it is likely that the contrasting RIP3 haplotypes segregating in the Jagger (3_SNPs) × 2174 (1_SNP) (Li et al. 2013) and Claire (3_SNPs) × Hereward (1_SNP) (Diaz et al. 2012) populations could have contributed to the differences in heading time observed after partial vernalization in these studies. This conclusion, does not rule out the possibility that the polymorphisms at position 180 or the differences in copy number could have also contributed to the observed differences in heading time in the previous studies.

The plants from the F_2_ population segregating for the RIP3 haplotypes have a single *VRN-A1* copy encoding identical proteins, but differ in *VRN-A1* transcript levels. Therefore, polymorphisms at the *VRN-A1* regulatory regions are good candidates to explain the differences in heading time linked to this gene. A comparison of the *VRN-A1* promoter regions (2254 bp upstream from the start codon) from the 1_SNP and 3_SNPs haplotypes revealed no-polymorphisms in the first 436 bp (Supplemental Figure S1). The rest of the promoter region (437–2254 bp) showed seven SNPs and four indels (1–2 bp), but none of them were located within known regulatory elements (Pidal et al. 2009; Kane et al. 2007; Li and Dubcovsky 2008; Li et al. 2015), predicted binding sites of transcription factors, or evolutionary conserved regions (Supplemental Figure S1). By contrast, the RIP3 polymorphisms have been shown to affect the binding of GRP2 proteins to the pre-m*VRN-A1* transcripts (Kippes et al. 2015), a result consistent with the differences in relative abundance of alternative splice variants described in the following section.

### Differences in *VRN-A1* expression are consistent with the proposed RIP3/GRP2 molecular mechanisms

Diaz et al. (2012) observed faster and higher *VRN-A1* transcript levels in Claire (3_SNPs haplotype) than in the two varieties carrying the 1_SNP haplotype. Re-analysis of the expression data from Li et al. (2013) using a two-way ANOVA with time (3 weeks and 6 weeks) and genotypes (Jagger and 2174) as factors, revealed higher *VRN-A1* transcript levels in Jagger (3_SNPs) than in 2174 (1_SNP, *P* < 0.0001, Supplemental Table S2). These results are similar to the ones presented here, and suggest that at least part of the differences in heading time between these two *VRN-A1* alleles are regulated at the transcriptional level.

We have recently shown that the natural polymorphisms found in the 3_SNPs haplotype result in reduced binding of the GRP2 protein to the RIP3 site in the *VRN-A1* first intron (Kippes et al. 2015). GRP2 has been previously shown to be a repressor of flowering that binds the pre-m*VRN-A1* transcript (Xiao et al. 2014). During vernalization, GRP2 is O-GlcNAc modified and its levels in the nucleus decrease, allowing higher *VRN1* mRNA accumulation (Xiao et al. 2014).

Differences in the speed and processivness of transcription affects the selection of alternative splicing sites (de la Mata et al. 2003). Therefore, the stronger binding of the GRP2 protein to the translated RIP3 sites with the 1_SNP haplotype may favor the *VRN-A1* short alternative splice variant, first described by Xiao et al. (2014). We detected in silico a short alternative splice variant for *VRN-B1*, which showed the same structure as the one described for *VRN-A1* (http://plants.ensembl.org, TGACv1, Traes_5BL_89636D032.1). However, we did not find this short variant in the D genome of Chinese Spring, a result that is consistent with a deletion encompassing the RIP3 site in the first intron of *VRN-D1*.

The binding of the GRP2 protein to the pre-mRNA RIP3 site may explain the significantly higher short/long variant ratio observed before vernalization in the F_2_ plants carrying the 1_SNP haplotype relative to those carrying the 3_SNPs haplotype in this study (Fig. 2d). The reduced GRP2 levels during and after vernalization would favor the long transcript variant and explain the decrease in the short/long variant ratio in these later time points. The higher relative abundance of the short splice variant before vernalization may contribute to maintain low levels of functional *VRN-A1* until the vernalization requirement is satisfied. Alternatively, the 155 amino acids encoded by the short variant (including the MADS-box domain MADS_MEF2_like, cd00265) may interact with other MADS-Box proteins altering their function. Transgenic experiments overexpressing this short variant will be required to test this hypothesis. Once the role of these alternative splice variants is better understood, it may provide an additional entry point to modulate the vernalization requirement in wheat.

Based on the previous results and discussion, we favor the hypothesis that the polymorphisms in the RIP3 region are responsible for the differences in *VRN-A1* transcript levels and heading time between the lines carrying the 1_SNP and 3_SNPs haplotypes. However, we cannot rule the possibility of effects caused by linked polymorphisms in the promoter region or outside the sequenced region.

### Winter wheat varieties carrying the 3_SNPs haplotype were detected at low frequencies

A recent study of 1,100 winter wheat lines from different regions of the world (with emphasis in European varieties) found a single *VRN-A1* copy in only 7% of the varieties (Würschum et al. 2015). This percentage is similar to the 10.2% of varieties with a single *VRN-A1* copy found in this study. Unfortunately, Würschum et al. (2015) did not have information about the RIP3 haplotypes.

Since 92.3% of the accessions with a single *VRN-A1* copy in our winter panel have the 3_SNPs haplotype, we will assume for the following discussion that in the winter panel from Würschum et al. (2015) most of the accessions carrying a single *VRN-A1* copy also carry the 3_SNPs allele. Würschum et al. (2015) found that the frequency of the varieties with a single *VRN-A1* copy was larger in Southern Europe and the UK where the winters are milder (Würschum et al. 2015). This distribution is consistent with the milder vernalization requirement of the varieties carrying the 3_SNPs haplotype. We present the geographical origins of 12 accessions with 3_SNPs haplotypes detected in this study in Supplemental Table S4, but due to the small sample size, it is difficult to draw any solid conclusion.

The only accession in this survey with one *VRN-A1* copy and the 1_SNP haplotype (PI 627798) was the latest or second latest flowering line when compared with the accessions with a single *VRN-A1* copy and the 3_SNPs haplotype, and within the range of the accession with multiple *VRN-A1* copies and the 1_SNP haplotype. The late flowering of PI 627798 is also consistent with the late flowering of the plants carrying the 1_SNP haplotype in our F_2_ population, which suggests that the RIP3 haplotypes correlate better with heading time in winter wheat than the differences in *VRN-A1* copy number.

### *VRN-A1* copy number variants within the 1_SNP haplotype showed limited association with differences in heading time

**Fig. 5:**
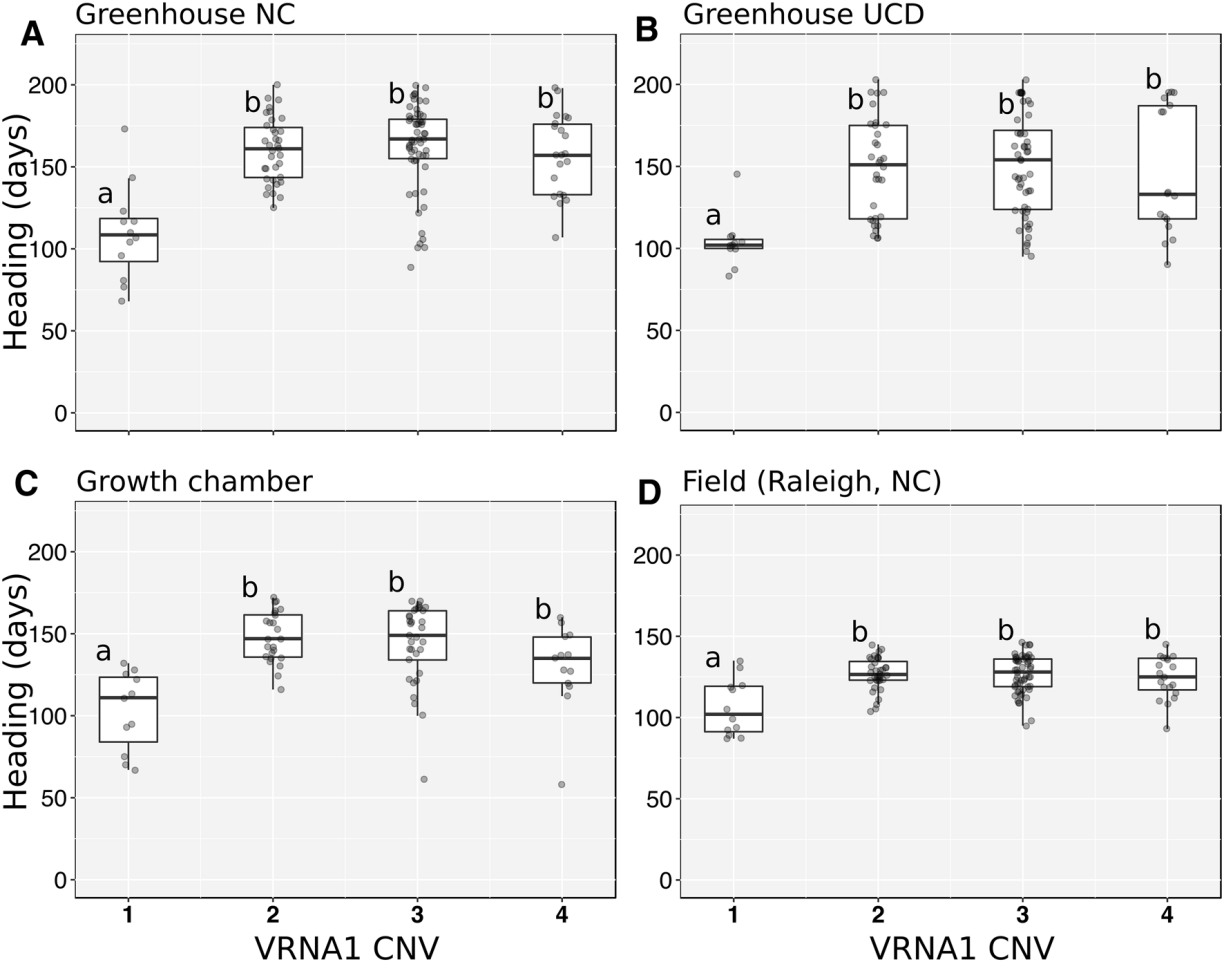
Heading time differences among winter wheats with different *VRN-A1* copy number. The relative copy number and *VRN-A1* haplotypes are described in supplemental Table S4. Lines were grouped based on the estimated *VRN-A1* copy number and average heading times among groups were compared using a Tukey’s test. Different letters above the box-plots indicate significant differences among groups (*P* < 0.05)

Once we removed the effect of the 3_SNPs haplotype, we did not detect differences in heading time among the winter wheat varieties with two, three or four *VRN-A1* copies in any of the field or controlled environment experiments with partial vernalization (Fig. 5). Even the single variety found with one *VRN-A1* copy with the 1_SNP haplotype (PI 627798) flowered within the range of the varieties with multiple *VRN-A1* copies.

The study by Würschum et al. (2015) also failed to detect differences in heading time among varieties with two, three or four *VRN-A1* copies. However, their heading time studies were performed under field conditions in Germany (> 48°N), where winter conditions were likely sufficient to satisfy completely the vernalization requirement. We cannot rule out a role of *VRN-A1* copy number under different conditions. In fact, in a double haploid population from the cross between Malacca (two *VRN-A1* copies) and Hereward (three *VRN-A1* copies) subjected to partial vernalization, Diaz et al. (2012) showed that the lines with three *VRN-A1* copies flowered later than those with two copies. Guedira et al. (2016) observed that RILs from the AGS 2000 × 26R61 RIL population with two *VRN-A1* copies flowered earlier than the lines with three copies when the population was grown in the field at locations with mild winters in the southeastern United States. It would be interesting to test if the same effect can be detected in other biparental populations segregating for *VRN-A1* copy number variants.

Although the frequencies of varieties with different *VRN-A1* copy number alleles in different geographical regions suggest a possible adaptive role, additional studies using isogenic lines or biparental populations will be necessary to quantify better the effect of the number of *VRN-A1* copies on the adaptation to different environments. Adaptation to these environments may depend not only on the effect of the different *VRN-A1* copy number variants on heading time, but also in their interactions with *FR-A2* alleles for frost tolerance. Zhu et al. (2014) showed that winter wheat varieties carrying three *VRN-A1* copies were more frost tolerant than varieties with two *VRN-A1* copies when the *FROST TOLERANCE 2* allele *T* (*FR-A2-T*) was present.

## Conclusions and practical applications

It is still not clear if the relatively low frequency of the 3_SNPs allele in the Western breeding programs is a result of its recent introduction or the effect of a narrow adaptive value, limited to a small range of environments. If the 3_SNPs allele introgression happened recently, it is possible that this *VRN-A1* allele has not reached yet its optimal frequency. It would be interesting to test the value of this allele in modern winter wheat breeding programs, particularly in regions with mild winters. It is also possible, that as global temperatures increase and winters become milder, the 3_SNPs allele will became more valuable in certain regions.

The presence of the 3_SNPs haplotype in the successful winter wheat variety Jagger may represent an example of the potential of this allele. Jagger was grown in more than 25% of the Oklahoma acreage for 11 years and was the dominant wheat variety in Kansas between 1998 and 2010 (USDA/NASS Oklahoma Field Office wheat.okstate.edu and kswheat.com). Although we do not know how much the 3_SNPs *VRN-A1* haplotype contributed to Jagger success, it would be interesting to characterize the presence of this allele in the multiple varieties derived from Jagger. Additionally, it will be informative to monitor the changes in the 3_SNPs allele frequency as new varieties are released in this region.

In summary, we have shown a significant effect of the RIP3 haplotypes on wheat heading times, both under controlled environments with partial vernalization and in field experiments. Our results and those from Würschum et al. (2015) suggest that one *VRN-A1* copy with the 3_SNPs haplotype may have an adaptive value in regions with mild winters. The confirmation of the role of the 3_SNPs allele on heading time and in the modulation of the vernalization requirement can provide winter wheat breeders new genetic tools to improve wheat adaptation to new or changing environments.

## Electronic supplementary material

Below is the link to the electronic supplementary material.

Supplementary material 1 (DOCX 18 KB)

Supplementary material 2 (DOCX 21 KB)

Supplementary material 3 (XLSX 19 KB)

## Abbreviations

*FT1*: *FLOWERING LOCUS T1*
GRP2: GLYCINE RICH RNA-BINDING PROTEIN 2
RIP3: RNA immune precipitation fragment 3
SNP: Single nucleotide polymorphism
*VRN1*: *VERNALIZATION1* gene
*VRN2*: *VERNALIZATION2* gene
*VRN3*: *VERNALIZATION3* gene
*VRN-D4*: *VERNALIZATION4* gene D genome
