# Supplementary material for: Single nucleotide polymorphisms in a regulatory site of VRN-A1 first intron are associated with differences in vernalization requirement in winter wheat
Source: Mol Genet Genomics. 2018 Jun 5;293(5):1231–43. doi: 10.1007/s00438-018-1455-0 (PMC6153499; doi:10.1007/s00438-018-1455-0)
Supplement: Supplementary file 1 — Supplementary material 1 (DOCX 18 KB) [file 438_2018_1455_MOESM1_ESM.docx]

**Supplementary Figure S1**. Comparison of *VRN1* promoter regions of TDC (1_SNP haplotype) and CS5402 (2_SNP haplotype). SNPs are highlighted in yellow and indels in black.

>TDC *VRN-A1* promoter 2,254 bp. (GeneBank MH347747)

ATTTGTATTGACTATTTGATTTCTATTGATTTTCTATGATGAACT**ACGT**GACAAAAAAATAGCTTCTGTTGAATTTGTGCCGAAGCACGCTGAATACCGGCCGATAGTGGGCCAATTTGCGCCGATAGTGGGGTAATTTGCGCCGAAAATGAGCTGAAAAGTGGGCCAATTTGCACTGAAAGTGGGCCAAAATCAGCACCTGAGGACGGCGGCTGGGAACCAGATCGCCCCCAGGCCGATATTTCAGCCGGCACTCCCCCAGGACGGCGCTATTTCTAGCCCCTGGGGGCCGAACGAGTGGAGATGTTCTAACAATTGTGAAAACTTAGCTTAGATAATAGGTAGGTCCATATACATGCAAAATTTGCAATAAAAGGATCATCCGTGAGCTAAAGATAAATAGGAAACCAAGTAATCACTAACTTGCCTGAAATAGTCTTTAAGTTACAGTAAACTAGAAATTATTATATCTGCTATACCCATTTTTTCACAAGCATTTGGAACTCTGACATGTTTTTGAAGTACTGATTATATAGAGTGCGTGTGATCTAAATATCGTTTTTGCATTCTTGGAGATACACCGCAAAGTTTAGGGATGTTCATGTTTGTATATTTTCAATCGCAGAGATGTTTCGAATTAGTTGTCTTACATTTGTCTAGATATAGTATCTAGAC**ACGT**TTTAGTGTCAGATATCTCGGTATCTAGACAAATTTAAGATAAATAATTTGGGACGGAGTTAATACATGGCATATTTGCAGAATAAAGAAAAAGAAAAAAAGATGTACATGGCATATTCGCAAAGAAAAAAAAGAAAAGAAAAGAGATGTGCATGATATGATTGCGAGATCTGTCATCATCGGTCATAAAGAGCAGCATTGGTGGTTGCTGGTT**ACGT**CAAATGGTTGCAAAGTGGTCATTTGACAAGCATGCATATGGGCTTTTTGTTTGTTTGTCAGCATAAACAATAAAATGGGTCATGATTCACATGTTCGAACACTGAACTACTAAATGTTATATTAGGCCCAACAACAGTTTTAGTGTAGAAACCATTTTAGTGCTGCAAATGGTTTCAGCACGGCCCTATATATCGAGAAGCTGGTCACTGCAGAAGTGCAGATATATCACATGTACAGTTCTCTATAGAGCGTTTTTTTTGTCCATATATAAAGAAAAATACACGAACCGTTTCTGGAGTATATAATTGGTATGGAGTCGGACAACCCCACGATGAGACGCTTGACAACAGTGTATTGATGGATGGCTGGTCGGTATACACGCACAGCACAGTACCCCTACTCCTAGGACTGGCGAGTATCTTTCATTCATTCCAGAAATACGCGGGTCGGCCAAAAGTAGAAAAATACACTGCGCCCACTCGATCCACGCAGCGCACTGCACTGCACTGCACAGCAACGCTTCATGTCAAAAGTCGAGCTCAATCATGCACACGATGGACGCGGCGCGAACGACCCGGGCGGCCCGCCGCGCCCGCCCGCCCGCCCCGCAGCCGACCTCTCCCAAACGGGGCAAGCGAGACGGCCCAAAACGAGCAAGGAAAGCAGCCTCCTACTGTGGCAGCCCGCCCCCACGACCGTCATCTCGCCTTCCATTCCATTTTCCCTGGACGGACCAGACCCGTCCCGAGCCGCCCTGACCTAGCCAGCCAGCATTTCCTCTTTCGTCCCCCGCCGCCGTGACCAAAAAAGCAAAAAAGGAAAAAGGGGAAATGCTAAAGGAAAAAACTCCGCTCTTTCCCTTCTTCTAGGCCTAGGGTACAGTAGAATATTATGAAAGGAAAAATTCTGCTCGTTTTTTTTCTCTGTGGTGTGTGTTTGTGGCGAGAGAAAATGATTTGGGGAAAGCAAAATCCGGAGATTCGC**ACGT**ACGATCGTTCGAC**ACGT**CGACGCCCGGCGGGCCCGGGGTGGGGCATCGTGTGGCTGCAGGACCGCGGGGCCCCGCAAAGCGGGCCGGGCCAATGGGTGCTCGACAGCGGCTATGCTCCAGACCAGCCCGGTATTGCATACCGCGCTCGGGGCCAGATCCCTTTAAAAACCCCTCCCCCCCTGCCGGAATCCTCGTTTTGGCCTGGCCATCCTCCCTCTCCTCCCCTCTCTTCCACCTCACGTCCTCACCCAACCACCTGATAGCCATGGCTCCGCCGCCTCGCCTCCGCCTGCGCCAGTCGGAGTAGCCGTCGCGGTCTGCCGGTGTTGGAGGGTAGGGGCGTAGGGTTGGCCCGGTTCTCGAGCGGAGATG

>Chinese Spring *Vrn-A1* promoter (= CS5402) 2,251 bp (NRGene/URGI 0.4 Pseudomolecule Chr 5A 587425491-587423241)

ATTTGTATTGAACTATTTGATTTCTATTGATTTTCTATGATGAACT**ACGT**GACAAAAAAATAGCTTCTGTTGAATTTGTGCCGAAGCACGCCGAATACCGGCCGATAGTGGGCCAATTTGCGCCGATAGTGGGGTAATTTGCGCCCAAAATGAGCTGAAAAGTGGGCCAATTTGCACTGAAAGTGGGCCAAAATCAGCACCTGAGGACGGCGGCTGGGAACCAGATCGCCCCCAGGCCGATATTTCAGCCGGCACTCCCCCAGGACGGCGCTATTTCTAGCCCCTGGGGCCGAACGAGTGGAGATGTTCTAACAATTGTGAAAACTTAGCTTAGATAATAGGTAGGTCCATATACATGCAAAATTTGCAATAAAAGGATCATCCGTGAGCTAAAGATAAATAGGAAACCAAGTAATCACTAACTTGCCTGAAATAGTCTTTAAGTTACAGTAAACTAGAAATTATTATATCTGCTATACCCATTTTTTCACAAGCATTTGGAACTCTGACATGTTTTTGAAGTACTGATTATATAGAGTGCGTGTGATCTAAATATCGTTTTTGCATTCTTGGAGATACACCGCAAAGTTTAGGGATGTTCATGTTTGTATATTTTCAATCGCAGAGATGTTTCGAATTAGTTGTCTTACATTTGTCTAGATATAGTATCTAGAC**ACGT**TTTAGTGTCAGATATCTCGGTATCTAGACAAATTTAAGATAAATAATTTGGGACGGAGTTAATACATGGCATATTTGCAGAATAAAGAAAAAGAAAAAAGATGTACATGGCATATTCGCAAAGAAAAAAAAGAAAAGAAAAGAGATGTGCATGATATGATTGCGAGATCTGTCATCATCGGTCATAAAGAGCAGCATTGGTGGTTGCTGGTT**ACGT**CAAATGGTTGCAAAGTGGTCATTTGACAAGCATGCATATGGGCTTTTTGTTTGTTTGTCAGCATAAACAATAAAATGGGTCATGATTCACATGTTCGAACACTGAACTACTAAATGTTGTATTAGGCCCAACAGCAGTTTTAGTGTAGAAACCATTTTAGTGCTGCAAATGGTTTCAGCACGGCCCTATATATCGAGAAGCTGGTCACTGCAGAAGTGCAGATATATCACATGTACAGTTCTCTATAGAGCGTTTTTTTTCTCCATATATAAAGAAAAATACACGAACCGTTTCTGGAGTATATAATTGGTATGGAGTCGGACAACCCCACGATGAGACGCTTGACAACAGTGTATTGATGGATGGCTGGTCGGTATACACGCACAGCACAGTACCCCTACTCCTAGGACTGGCGAGTATCTTTCATTCATTCCAGAAATACGCGGGTCGGCCAAAAGTAGAAAAATACACTGCGCCCACTCGATCCACGCAGCGCACTGCACTGCACTGCAGCAACGCTTCATGTCAAAAGTCGAGCTCAATCATGCACGCGATGGACGCGGCGCGAACGACCCGGGCGGCCCGCCGCGCCCGCCCGCCCGCCCCGCAGCCGACCTCTCCCAAACGGGGCAAGCGAGACGGCCCAAAACGAGCAAGGAAAGCAGCCTCCTACTGTGGCAGCCCGCCCCCACGACCGTCATCTCGCCTTCCATTCCATTTTCCCTGGACGGACCAGACCCGTCCCGAGCCGCCCTGACCTAGCCAGCCAGCATTTCCTCTTTCGTCCCCCGCCGCCGTGACCAAAAAAGCAAAAAAGGAAAAAGGGGAAATGCTAAAGGAAAAAACTCCGCTCTTTCCCTTCTTCTAGGCCTAGGGTACAGTAGAATATTATGAAAGGAAAAATTCTGCTCGTTTTTTTGCTCTGTGGTGTGTGTTTGTGGCGAGAGAAAATGATTTGGGGAAAGCAAAATCCGGAGATTCGC**ACGT**ACGATCGTTCGAC**ACGT**CGACGCCCGGCGGGCCCGGGGTGGGGCATCGTGTGGCTGCAGGACCGCGGGGCCCCGCAAAGCGGGCCGGGCCAATGGGTGCTCGACAGCGGCTATGCTCCAGACCAGCCCGGTATTGCATACCGCGCTCGGGGCCAGATCCCTTTAAAAACCCCTCCCCCCCTGCCGGAATCCTCGTTTTGGCCTGGCCATCCTCCCTCTCCTCCCCTCTCTTCCACCTCACGTCCTCACCCAACCACCTGATAGCCATGGCTCCGCCGCCTCGCCTCCGCCTGCGCCAGTCGGAGTAGCCGTCGCGGTCTGCCGGTGTTGGAGGGTAGGGGCGTAGGGTTGGCCCGGTTCTCGAGCGGAGATG

**Polymorphisms**

SNPs

Indels

**Regulatory regions**

ATG: start codon.

Green letters: 5’ UTR (by 5’ RACE).

CCTCGTTTTGG: Conserved CArG box. VRT2 binding site by electrophoretic mobility shift assay.

**ACGT**: ACGT-box. FD2 binding site by electrophoretic mobility shift assay.

CTTTAAAAACCCC: Conserved vernalization-box. Predicted by natural deletions, includes TATA-box.

CCGAC: predicted CRT/DRE core motif recognized by CBF transcription factors.

GTACGA: conserved SPL binding site (predicted).

TCGACAGC: Conserved ARF binding site (predicted).

CCAAT: Conserved CAT box (predicted).
