# Supplementary material for: Single nucleotide polymorphisms in a regulatory site of VRN-A1 first intron are associated with differences in vernalization requirement in winter wheat
Source: Mol Genet Genomics. 2018 Jun 5;293(5):1231–43. doi: 10.1007/s00438-018-1455-0 (PMC6153499; doi:10.1007/s00438-018-1455-0)
Supplement: Supplementary file 2 — Supplementary material 2 (DOCX 21 KB) [file 438_2018_1455_MOESM2_ESM.docx]

| **Table S1.** Description of KASP markers for alleles conferring spring growth habit used to genotype accessions from the USDA National Germplasm Collection. | | | | |  |
| --- | --- | --- | --- | --- | --- |
| **Locus** | **Allele(s) assayed** | **Marker ID** | **Primer name** | **Primer Sequence** | **Reference^1^** |
| Vrn-A1 | Vrn-A1a | wMAS000033 | Vrn-A1_9K0001_AL2 | GAGTTTTCCAAAAAGATAGATCAATGTAAAC | Cavangah et al. 2013 |
|  |  |  | Vrn-A1_9K0001_AL1 | AGAGTTTTCCAAAAAGATAGATCAATGTAAAT |  |
|  |  |  | Vrn-A1_9K0001_C1 | GTTAGTAGTGATGGTCCAATAATGCCAAA |  |
|  | Vrn-A1b | wMAS000035 | Vrn-A1b-Marq_AL2 | GTTTTGGCCTGGCCATCCTCA | Yan et al. 2004 |
|  |  |  | Vrn-A1b-Marq_AL1 | GTTTTGGCCTGGCCATCCTCC |  |
|  |  |  | Vrn-A1b-Marq_C1 | TATCAGGTGGTTGGGTGAGGACGT |  |
|  | vrn-A1 exon 4_C/T | vrn-A1exon4 | Vrn-A1_Exon4_F1 | AGGCATCTCATGGGAGAGGATC | Diaz et al. 2012 |
|  |  |  | Vrn-A1_Exon4_F2 | CAGGCATCTCATGGGAGAGGATT |  |
|  |  |  | Vrn-A1_Exon4_R | CCAGTTGCTGCAACTCCTTGAGATT |  |
| Vrn-B1 | Vrn-B1a | Vrn-B1_I_D | Vrn-B1_D_A2 | GGCAGCTAATGTGGGGTAGTCT | Fu et al. 2005 |
|  |  |  | Vrn-B1_D_C1s | ATTCGTATTGCTAGCTCCGGCCAT |  |
|  |  |  | Vrn-B1_I_ALG | CAACCTCCACGGTTTCAAAAAGTAG |  |
|  |  |  | Vrn-B1_I_C1 | ATATTTACTAAGCAGCGGTCATTCCGAT |  |
|  | Vrn-B1b | wMAS000037 | Vrn-B1_B_ALC | GCGCAAGCGGGAGCTACATC | Santra et al. 2009 |
|  |  |  | Vrn-B1_B_ALG | TGCGCAAGCGGGAGCTACATG |  |
|  |  |  | Vrn-B1_B_C1 | GCCATGAACAACAAAGGGGGTGGT |  |
|  | Vrn-B1c | Vrn-B1_C | Vrn-B1_C _ALT | CCTAAACAGGGGCAGAACACTA | Milec et a. 2012 |
|  |  |  | Vrn-B1_C _ALG | CCTAAACAGGGGCAGAACACTG |  |
|  |  |  | Vrn-B1_C _C | GACCCCAGGGCCTATGAATGTAATT |  |
|  | vrn-B1_intron1_A/C | TaVrn-B1_1752 | TaVrnB1_1752_AF2 | GGAATGACCGCTGCTTAGTAAATATA | Guedira et al. 2014 |
|  |  |  | TaVrnB1_1752_CF1 | GGAATGACCGCTGCTTAGTAAATATC |  |
|  |  |  | TaVrnB1_1752_R | GATTTAGCACCTCAACATACAGGTCT |  |
| Vrn-D1 | Vrn-D1a | wMAS000039 | Vrn-D1-D1a_A_ALC | ATCATTCGAATTGCTAGCTCCGC | Fu et al. 2005 |
|  |  |  | Vrn-D1-D1a_A_ALG | ATCATTCGAATTGCTAGCTCCGG |  |
|  |  |  | Vrn-D1-D1a_A_C | GCCTGAACGCCTAGCCTGTGTA |  |
| Vrn-B3 | Vrn-B3 | Vrn-B3_Hope | Vrn-B3_1_A1 | GAGTAATTAAAGCTCGATCTTAAATACTCT | Yan et al. 2006 |
|  |  |  | Vrn-B3_1_A2 | CGAGTAATTAAAGCTCGATCTTAAATACTTC |  |
|  |  |  | Vrn-B3_1_C2 | GACTTAAGACAAAAGCTAGAAGTACATTTA |  |

^1^Reference for previously published KASP assay or sequence used for assay design. KASP assays do not include tail sequences.

**Table S2**. Transcript levels of *VRN-A1* relative to *ACTIN* calculated using the 2^(-ΔΔCT)^ method, where CT is the threshold cycle ^1^.

| Genotype | 3 weeks vernalization | 6 weeks vernalization |
| --- | --- | --- |
|  | 46.4 | 243.5 |
|  | 33.0 | 356.1 |
|  | 28.7 | 354.4 |
| Jagger | 42.1 | 370.6 |
|  | 44.0 | 134.9 |
|  | 21.1 | 850.2 |
|  | 29.6 | 111.1 |
|  | 8.7 |  |
| Average  SE of the mean | **31.7**  4.5 | **345.8**  93.3 |
|  | 5.9 | 37.1 |
|  | 3.7 | 54.7 |
|  | 7.1 | 202.5 |
|  | 8.9 | 33.2 |
| 2174 | 9.1 | 57.1 |
|  | 10.4 | 15.7 |
|  | 35.6 | 285.1 |
|  | 8.7 | 297.6 |
|  | 11.4 | 137.9 |
|  | 6.5 | 205.9 |
|  | 6.5 | 43.6 |
| Average  SE of the mean | 10.3  2.6 | 124.6  32.0 |
| 2-way ANOVA | F-value | *P* |
| Genotype  Time  Genotype * Time | 23.8  84.1  0.0 | <0.0001  <0.0001  0.9541 |

^1^ Data generously provided by Dr. Liuling Yan, Oklahoma State University

**Table S3.** Primers used for Sanger based sequencing of Triple Dirk C *VRN-A1* promoter.

| **Forward** | | **Reverse** | | **Size (bp)** |
| --- | --- | --- | --- | --- |
| F1 | GCCTAGTTCGACGTGTGATG | R1 | CTTCTGCAGTGACCAGCTTC | 1152 |
| F2 | CACCGCAAAGTTTAGGGATGT | R2 | GAGTGGGCGCAGTGTATTTT | 799 |
| F3 | CAGCACGGCCCTATATATCG | R3 | AGAAGAAGGGAAAGAGCGGA | 691 |
| F3 | CCAGCCAGCATTTCCTCTTT | R3 | GACGTGAGGTGGAAGAGAGG | 468 |
